# Wheat (*Triticum aestivum* L.) *TaHMW1D* Transcript Variants Are Highly Expressed in Response to Heat Stress and in Grains Located in Distal Part of the Spike

**DOI:** 10.3390/plants10040687

**Published:** 2021-04-02

**Authors:** Chan Seop Ko, Jin-Baek Kim, Min Jeong Hong, Yong Weon Seo

**Affiliations:** 1 Department of Plant Biotechnology, Korea University, 145 Anam-ro, Seongbuk-gu, Seoul 02841, Korea; chansubi@korea.ac.kr; 2 Advanced Radiation Technology Institute, Korea Atomic Energy Research Institute, 29 Geumgu, Jeongeup 56212, Korea; jbkim74@kaeri.re.kr (J.-B.K.); hongmj@kaeri.re.kr (M.J.H.)

**Keywords:** wheat, high-molecular glutenin gene, transcript variants, high temperature stress, grain-filling period, spikelet position in spike

## Abstract

High-temperature stress during the grain filling stage has a deleterious effect on grain yield and end-use quality. Plants undergo various transcriptional events of protein complexity as defensive responses to various stressors. The “*Keumgang*” wheat cultivar was subjected to high-temperature stress for 6 and 10 days beginning 9 days after anthesis, then two-dimensional gel electrophoresis (2DE) and peptide analyses were performed. Spots showing decreased contents in stressed plants were shown to have strong similarities with a high-molecular glutenin gene, *TraesCS1D02G317301* (*TaHMW1D*). QRT-PCR results confirmed that *TaHMW1D* was expressed in its full form and in the form of four different transcript variants. These events always occurred between repetitive regions at specific deletion sites (5′-CAA (Glutamine) GG/TG (Glycine) or (Valine)-3′, 5′-GGG (Glycine) CAA (Glutamine) -3′) in an exonic region. Heat stress led to a significant increase in the expression of the transcript variants. This was most evident in the distal parts of the spike. Considering the importance of high-molecular weight glutenin subunits of seed storage proteins, stressed plants might choose shorter polypeptides while retaining glutenin function, thus maintaining the expression of glutenin motifs and conserved sites.

## 1. Introduction

The quality and quantity of wheat, the world’s most important cereal crop for human consumption, are affected by environmental stress, genotype, and interactions between the two [1]. Climate is a predominant uncontrollable factor influencing crop yield [2]. Heat stress, especially that experienced during the grain-filling stage, significantly affects grain yield and end-use quality [3,4]. High-temperature stress affects photosynthesis ability [5] and causes the accumulation of reactive oxygen species, leading to cell death [6,7,8].

A wheat spike contains a variable number of spikelets, each with several florets [9]. Grains from different spikes and spikelets have different phenotypic appearances and developmental stages [10]. Grains from the central spikelets are heavier than grains from distal parts of the spike under heat stress [11]. The degree and rate of grain filling on a given spikelet are highly dependent on their positions in the spike. Thus, spikelet position in a spike affects total grain quality under heat stress.

Among wheat storage proteins, glutenin is largely responsible for the properties of wheat flour dough. The quality and quantity of glutenin significantly determine the elasticity and bread-making quality of a dough [12]. Polymeric glutenin comprises high-molecular weight and low-molecular weight glutenin subunits, the relative quantities and types of which are associated with the qualities of different wheat cultivars [13]. High-molecular weight glutenin subunits (HMW-GS) ranging from ~65 to 90 kDa [14] are encoded by genes located near the centromere of the long arm of group 1 chromosomes 1A, 1B, and 1D (*Glu-A1*, *Glu-B1*, and *Glu-D1*) [12]. HMW-GS composition is closely associated with bread-making quality [15], which is strongly influenced by the macropolymer formation of disulfide bonds in HMW-GS and low-molecular weight glutenin subunits (LMW-GS) as well as gliadin [16]. LMW-GS account for about 60% of total glutenin content [17]. LMW-GS is associated with dough strength and extensibility [18]. Studies of the allelic variation in glutenin subunits and the relationships between different types of glutenin have led to the establishment of successful breeding strategies [19]. Glutenins are essential proteins for baking because gluten polymerization leads to the development of networks.

Abiotic stresses during the grain filling stage can reduce wheat yield and quality. Environmental factors influence the composition of the reserve proteome in a way that varies depending on the genotype [20]. The end-use quality of the wheat was previously shown to be impacted by high temperatures during the grain filling phase [21]. In particular, the expression of glutenin subunits, which are crucial to wheat end-use quality, determines the parameters that could be significantly affected by heat stress. Heat stress diminishes flour quality by reducing gluten strength [22]. Since the expression of glutenin macropolymers is reduced by heat stress in the early grain filling stage [23], an analysis of glutenin expression in response to heat stress would be helpful to understand the negative effects of heat stress on wheat storage protein quality.

HMW-GS were composed of three specific domains, conserved (N- and C- terminal domains) and repetitive domains [24]. Repetitive domains, consisting of hexapeptide, nonapeptide and tripeptide motifs, were variable because of slippage events [25]. Slippage events in repetitive domains have contributed to exon duplication or deletions [26]. Repetitive regions have been linked to functional domains due to slippage events to perform roles in different cellular processes that provide adaptation and resistance in plants under abiotic environmental stresses [27]. As such, it could be seen that the repetitive proteins in HMW glutenin were also easily variably changed by stresses during DNA replication.

Transcript variants provided a single gene with multiple functional mRNAs and create protein complexity [28]. In the case of humans, there have been several studies about the available function of transcript variants. Multiple mode of transcript variants produced various types such as full-length, deleterious gain-of-function or functional truncated protein [29]. The transcript variants of proteasome activator 28γ, which were included conserved residues and activation loop, regulated the cell cycle and apoptosis [30]. Hepatocyte nuclear factor-1-beta transcript variants with different functions were characterized at specific expression levels by different tissues or diseases [31]. Plants also might adapt to adverse environments by regulating gene expression under abiotic stresses. This phenomenon renders plants resistant to abiotic stresses by creating transcript variants. Transcript variants, encoding various protein isoforms, must be studied using the complete wheat genome sequence, which has not yet been completely revealed, and the increased availability of genome information from other plants [32]. The transcript variants of 47 WRKY transcript factor genes responded to salt stress treatment in *Triticum aestivum* [33]. Under high-temperature stress, heat shock protein transcription factors (HSF), the main role of which is to maintain homeostasis in response to heat stress as chaperones, are known to cause alternative splicing. In *Triticum aestivum*, isoforms of *TaHsfA2* regulate various HSFA2 proteins which control protein homeosis under drought and heat combination stress [34]. Furthermore, the transcript variants of HSF are responsible for different biological functions. The HSF regulation pathways facilitate functional diversity under different biotic and abiotic stresses [35]. Dehydration and heat stresses are known to increase the amount of full length Serine/arginine rich like protein 45a relative to its other transcript variants in *Arabidopsis* [36]. Thus, plants might adapt to adverse environments by regulating gene expression via various transcripts variants under abiotic stresses.

The quality and quantity of HMW-GSs are considered as crucial parameters to determine end-use quality and their gene expressions are sensitive to heat stress. Plants might adapt to adverse environments by regulating gene expressions, of which expression variants such as transcript variants could be an example. Analysis of the decreased glutenin transcripts and proteins levels could be helpful to suggest that grains located at distal parts of the spike are significantly poorer than those reside in the middle part under heat stress. However, the mechanisms of molecular regulation of high-molecular glutenin genes in response to high-temperature stress during the grain filling period remain unclear. In this study, we determined that the glutenin gene exhibits decreased transcripts and protein levels because the plant employed various transcript variants mechanisms in response to high temperatures.

## 2. Results

### 2.1. Analysis of High-Molecular Weight Glutenin Subunits (HMW-GS)

**Figure 1:**
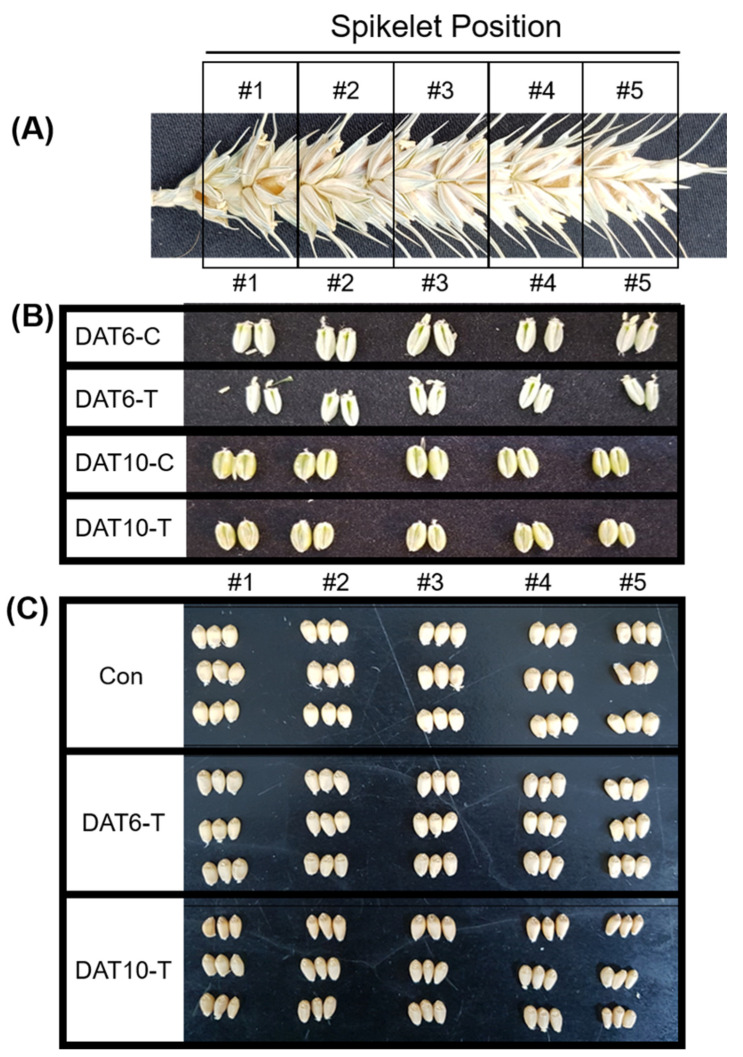
Spikelet position and seeds collected from spikes under high-temperature stress. (**A**) Division of a spike from base to top (spikelet #1–5). (**B**) Seeds taken from the five different sections of the spike. (**C**) Seeds from mature plants collected from the five different spike sections. Con, nontreated; DAT6-T, 6 days of treatment; DAT6-C, 6 days of nontreated control; DAT10-T, 10 days of treatment; DAT10-C, 10 days of nontreated control.

**Figure 2:**
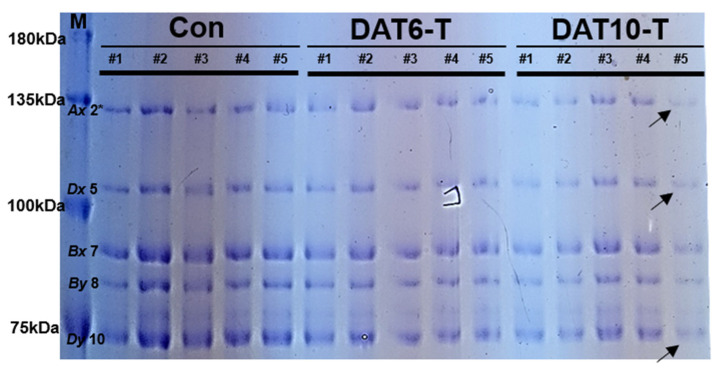
High-molecular weight glutenin subunit (HMW-GS) profiles of spikelets from different sections of the spike (#1–5, Figure 1A) of heat-treated plants. Glutenins were extracted from mature seeds harvested from high-temperature stress-treated wheat and nontreated control wheat. *Ax*, *Dx*, *Bx*, *By*, and *Dy* indicate HMW-GSs. M, Molecular size marker; Con, nontreated control; DAT6-T, 6 days of treatment; DAT10-T, 10 days of treatment.

HMW-glutenins were extracted from grain samples of heat-treated and control plants (Con; nontreated, DAT6-T; 6 days of treatment, DAT10-T; 10 days of treatment) at different spikelet positions (#1–5) (Figure 1). Total seed storage proteins (100 ng/µL) were separated by SDS-PAGE. All samples showed a defined group of bands ranging from 70 to 135 kDa corresponding to characteristic bands for HMW-GS. HMW-GS compositions including *Glu-A1* (subunit *Ax* 2*), *Glu-B1* (subunits *Bx* 7, *By* 8), and *Glu-D1* (subunits *Dx* 5, *Dy* 10) were observed. Heat stress-treated wheat lines exhibited altered HMW-GS profiles, as shown in Figure 2. The treated lines (DAT6-T & DAT10-T) showed significant decreases in the expression of all HMW-GSs compared to control plants (Con). The *Glu-A1* and *Glu-D1* subunits were less abundant than *Glu-B1*. Furthermore, HMW-GS production distinctly depended on the positions in the spike from which the grains were harvested from DAT10-T plants. The HMW-GS intensities from the grains at the spike edge (position #5) were significantly weaker than those at other positions (positions #2–4).

### 2.2. Gluten Analysis by 2DE-PAGE

**Figure 3:**
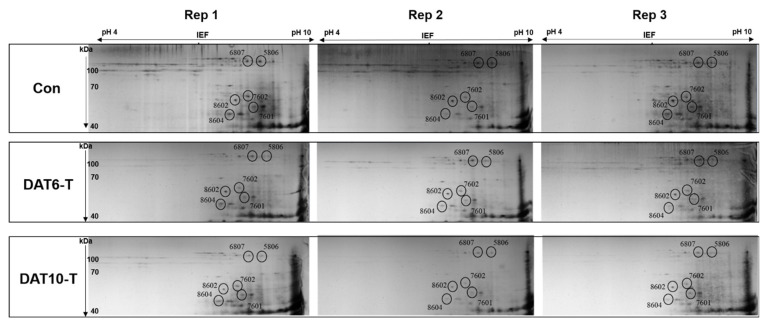
Identification of six glutenin spots with significantly decreased expression levels under high-temperature stress during grain filling. PI ranges are displayed at the top. Protein sizes are displayed to the left. Con, nontreated control; DAT6-T, 6 days of treatment; DAT10-T, 10 days of treatment. Three biological replicates were used (Rep. 1–3). The numbers near the circles indicate spot numbers.

Gluten proteins extracted from the total grains of Con, DAT 6-T, and DAT 10-T plants were analyzed by 2DE-PAGE (Figure S1 and Figure 3). A total of 111 spots were separated by 2DE (pI range of 4–10 for the first dimension and size range of 0–120 kDa for the second dimension). These protein spots were grouped by their expression patterns in the Con, DAT 6-T, and DAT 10-T samples (Figure S2, Table S1). The spots showing a unique pattern of decrease as heat treatment progressed (DAT6-T to DAT10-T) included six glutenins, 11 alpha/beta-gliadins, and 18 nongluten proteins classified by size. Of the spots that showed decreased contents in heat-stressed plants, six (Spot No. 5806, 6807, 7601, 7602, 8602, and 8604) were selected for further study (Figure 3).

### 2.3. Peptide Analysis of Selected Glutenin Spots

Analysis of these six spots revealed 40 protein pieces. The peptide information is shown in Table 1. DNA sequences derived from the sequences of these identified proteins were run through NCBI’s tBlastn (https://blast.ncbi.nlm.nih.gov/ (accessed on 22 January 2019)) and the Ensembl plant database (http://plants.ensembl.org/Multi/Tools/Blast (accessed on 20 November 2017)). These protein sequences showed strong similarities with *TraesCS1D02G317301*. Furthermore, all identified fractions were found to belong to the HMW-GS subunits, matching those from the homologous chromosomes *TraesCS1A02G317311* or *TraesCS1D02G317211*. The 1D-SDS and 2-DE analyses showed that *TraesCS1D02G317301* (*TaHMW1D*) expression decreased significantly during grain development in the heat stressed plants.

### 2.4. Sequence Analysis of TraesCS1D02G317301 (TaHMW1D)

**Figure 4:**
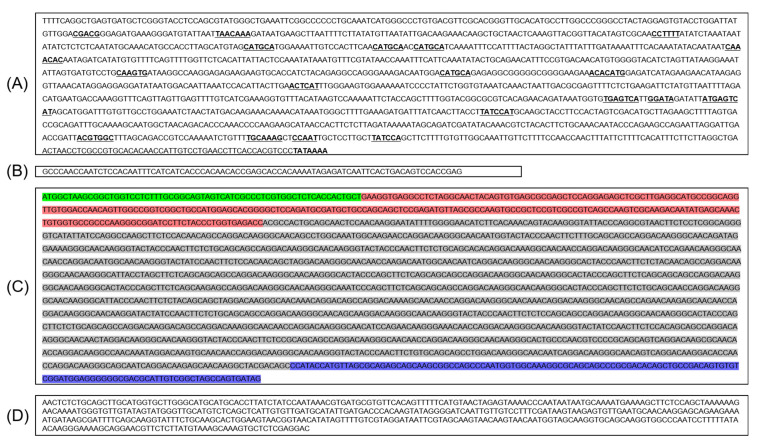
Complete *Keumgang TaHMW1D* gene sequence, including the (**A**) promoter, (**B**) 5′ UTR, (**C**) CDS, and (**D**) 3′ UTR. Bold and underlined, transcription factor binding sites; green, signal peptide region; pink, N-terminal region; gray, repetitive region; blue, C-terminal region.

To obtain comprehensive sequences of the promoter and open reading frame (ORF) regions of *TaHMW1D*, gene-specific primers were designed to amplify the 1.5 kb 5′-upstream region and 0.4 kb 3′-untranslated region. In c.v. *Keumgang*, the major transcription factor (TF) binding sites of *TaHMW1D* included a basic leucine zipper G-box binding factor (-CGACG-), N-ethylmaleimide-sensitive factor attachment protein gene promoters (-CAAACAC-), pyrimidine box TFs (-TAACAAA-), Storage protein enhancer box (-CAAGTG-), seed storage protein activator TFs (-ATGAGTCAT-), RY repeat motif (-CATGCA-), prolamin box TFs (-TGCAAAG-), pyrimidine box alpha amylase (-CCTTTT-), basic leucine zipper TFs (-ACACATG-), myeloblastosis family TFs (-GGATA-), arabidopsis thaliana basic leuncine-zipper2-binding site (-ACTCAT-), abscisic acid response (-ACGTGGC-), heat shock protein TFs (-CCAAT-), myeloblastosis-alpha-amylase gene TFs (-TATCCA-), and TATA box (-TATAAAA-) (Figure 4A).

Ensembl Plants provided limited CDS sequences for *TaHMW1D*, including numerous unknown nucleotides (*N*). To determine the exon and intron of *TaHMW1D*, PCR analyses were performed using genomic DNA. We isolated genomic sequences of *Keumgang TaHMW1D* using primers designed from the 5′ and 3′ UTRs (CAACCAATCTCCACAATTTCA and GTGGGTCATCAATATGCATCAACA, respectively) to amplify the *TaHMW1D* CDS region. In *Keumgang TaHMW1D*, the CDS contained a 1950-bp open reading frame encoding 650 amino acids (Figure 4C and Figure S3). The identified *TaHMW1D* gene was comprised of only one exon including a signal peptide, N-terminal regions, a repetitive domain, and C-terminal regions. The repetitive domain consisted of tandem repeats based on several peptide motifs (Table 2). The most frequent sequences were CAA (glutamine) and GGG (glycine).

**Figure 5:**
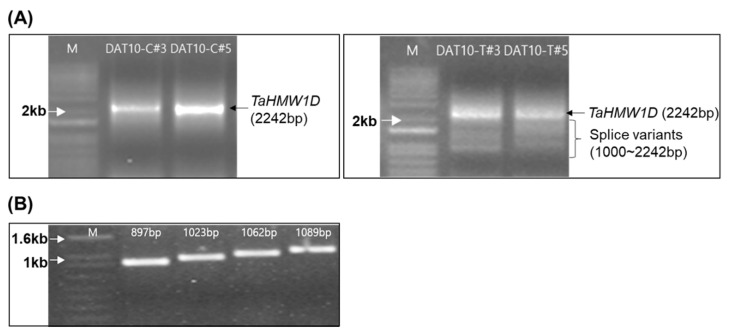
Identification of transcript variants. (**A**) Agarose gel (1%) showing the 2242-bp *TaHMW1D* gene product from control plants (left). The PCR products of high-temperature stress-treated wheat show smaller-sized (1000–2242 bp) transcript variants (right). (**B**) Sizes of the *TaHMW1D* transcript variants.

To isolate the translation region, PCRs were run using cDNAs prepared from DAT10-T#3, #5 and control plants (DAT10-C#3 and #5) with the same primers that were used to clone the ORF region of *TaHMW1D* gDNA. The PCRs revealed products of various sizes. The amount of the complete full-length transcript (2242 bp) was dramatically reduced in DAT10-T. Furthermore, various smaller transcripts were relatively more abundant in DAT10-T, indicating that transcript variants of a specific exon may have been produced by heat stress (Figure 5A). PCR products with lengths of 2242 bp (including UTRs and CDS) from treated and nontreated plants were identical to the *TaHMW1D* CDS, where 1950 bp was exactly the same as the previously cloned *TaHMW1D* gene sequence (Figure 4). Therefore, we concluded that *TaHMW1D* CDS consisted of only exonic DNA. The PCR products obtained from DAT10-T#3 and DAT10-T#5 were eluted, ligated with TA vector, and digested with *Eco*RI. Enzyme-digested fragments of various sizes from each clone were sequenced and four fragments were determined to be *TaHMW1D* transcript variants (Figure 5B). We were able to obtain one full-length gene (the full sequence of *TaHMW1D* with a length of 1950 bp) and four new cDNAs of different sizes (transcript variants of *TaHMW1D* with lengths of 897, 1023, 1062, and 1089 bp). Four numbers of *TaHMW1D* transcript variants, which were shorter than full length *TaHMW1D* gene, were identified.

### 2.5. Analysis of Transcript Variants

**Figure 6:**
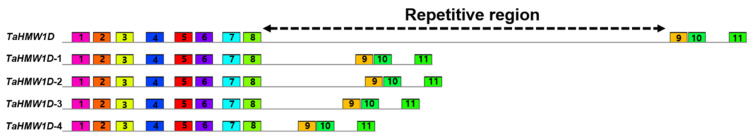
Conserved motif sequence alignments of *TaHMW1D* and its transcript variants in *Keumgang*. Motifs with identical sequences are given the same number. Signal peptide (1, 2), N-terminal region (3–8), C-terminal region (9–11). The region between motifs 8 and 9 is a repetitive sequence region in which deletion events occur.

**Figure 7:**
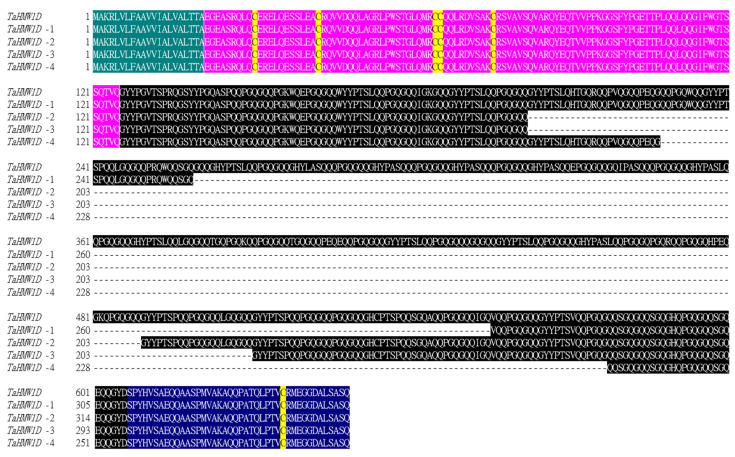
Protein sequence alignment of *TaHMW1D* and its transcript variants (*TaHMW1D*-1, 2, 3, 4) in *Keumgang.* Green, signal peptide; pink, N-terminal peptide; black, repetitive region; blue, C-terminal peptide. The conserved cysteine is marked in yellow.

**Figure 8:**
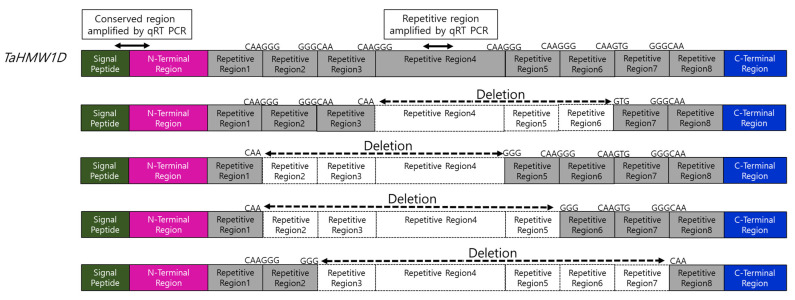
Features of *TaHMW1D* and its transcript variants *TaHMW1D*-1, 2, 3, and 4. qRT-PCRs amplifying either the conserved or repetitive regions are indicated. Specific deletion sites (CAAGG/TG GGGCAA) are indicated. Transcript regions that are deleted out during high-temperature stress are marked with empty boxes.

Eleven conserved motifs were detected in the complete *TaHMW1D* gene using Multiple EM for Motif Elicitation (MEME, http://meme-suite.org (accessed on 1 February 2020)) with generic parameters. All *TaHMW1D* transcript variants were aligned with the full-length *TaHMW1D*. A complete signal peptide (box 1–2), N-terminal (box 3–8), and C- terminal (box 9–11) regions were found in all transcript variants (Figure 6). These transcript variants and the full gene differed only between motifs 8 and 9, where repetitive motifs were present. Protein sequences from these transcript variants and the full CDS (Figure S4) were subjected to multiple sequence alignment using Clustal Omega (www.ebi.ac.uk/Tools/msa/clustalo (accessed on 1 October 2019)) (Figure 7). The motif and sequence analyses indicated that the *TaHMW1D* consisted of 8 different sizes of repetitive regions. These transcript variants had lengths of 1062 bp (*TaHMW1D*-1), 1089 bp (*TaHMW1D*-2), 1023 bp (*TaHMW1D*-3), and 897 bp (*TaHMW1D*-4). Differential transcript variants occurred in repetitive regions (Figure 8). Of the four transcript variants, *TaHMW1D*-4 had the largest deletion. Repetitive region 4 was absent from all transcript variants. The deletion events always occurred at a specific site between repetitive regions at the 3′ and 5′ specific deletion sites of either GG/TG (Gly) or CAA (Gln) in this experiment (Figure 8). The specific deletion sites were 5′-CAAGG/TG-3′, 5′-GGGCAA-3′, and the deletion events always occurred between CAA and GG/TG, which was frequently found in repeat motifs (Table 2).

Together, these results show that *Keumgang TaHMW1D* experienced deletion events in its repetitive region, always leaving conserved N- and C-terminal regions, during heat stress and grain filling. Cysteine residues were also conserved in the CDS of all transcript variants, indicating that they may maintain the characteristics of high-molecular-weight glutenins, in which intradisulfide bridges might form an important component of higher-order protein structure (Figure 7).

### 2.6. Expression Analysis of Transcript Variants Measured by qRT-PCR

**Figure 9:**
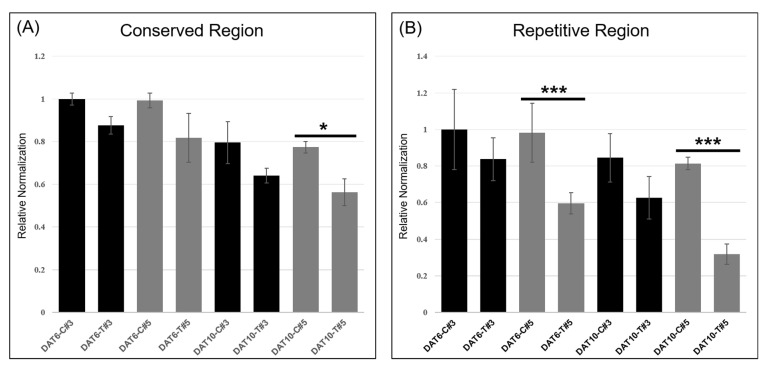
qRT-PCR of spikelets taken from different locations along the spike (position #3 or 5), targeting the conserved (**A**) or repetitive (**B**) region of *TaHMW1D*. * *p* < 0.05; *** *p* < 0.001.

qRT-PCR was performed to quantitatively evaluate the expression of *TaHMW1D* transcript variants, targeting the amplification of deleted (repetitive region 4, Figure 8, present only in full-length *TaHMW1D*) and conserved (signal peptide and N-terminal region, present in both *TaHMW1D* and its transcript variants, Figure 8) regions using grain samples from DAT6-C, DAT6-T, DAT10-C, and DAT10-T taken from different positions along the spike (#3 and 5, Figure 1A). We found that *TaHMW1D* expression (determined by the conserved region) significantly decreased as heat treatment progressed. The amounts of the transcripts in DAT10-T were lower than those in DAT6-T. In addition, the samples from spike position #5 had lower transcript contents than those from spike position #3 (Figure 9A). In order to analyze the expression of the transcript variants in response to prolonged heat stress as well as to identify spike-position-dependent expression, qRT-PCR was performed using primers targeting the intercalating region of repetitive region 4. We found that expression of this region also significantly decreased as heat treatment progressed. Furthermore, significant decreases in transcript amount were observed in DAT10-T and at spike position #5 compared to those in DAT6-T and at spike position #3, respectively (Figure 9B). Given the expression data of these two regions, we can conclude that heat stress induces a decrease in *TaHMW1D* transcript abundance and a corresponding increase in expression of the four transcript variants, *TaHMW1D*-1, 2, 3, and 4, and that these phenomena also occur in grains located at spike position #5.

## 3. Discussion

Since optimum allelic combinations of HMW-GS are crucial for improving the quality of bread, specific HMW-GS and LMW-GS are generally identified and selected using SDS-PAGE and 2DE methods [37]. Wheat flour quality, especially in terms of gluten strength [38] and viscoelasticity [39], is highly dependent on the quantity of seed storage proteins. Gluten constitutes approximately 85% of all endosperm proteins [40]. However, the mechanisms of molecular regulation of glutenin gene expression and protein synthesis in response to high-temperature stress during the grain filling period remain unclear.

We identified differences in expression of HMW-GSs in response to high-temperature stress during an early stage of grain filling using SDS-PAGE and 2DE. The *Glu-A1* and *Glu-D1* subunits were less abundant than *Glu-B1* following heat stress treatment (Figure 2). The *Glu-A1* and *Glu-D1* HMW subunits are associated with dough strength, breakdown ability, and gluten strength [41]. Many studies have investigated *Glu-A1* and *Glu-D1* in an effort to improve bread-making quality through creation of overexpression transgenic plants. Transgenic wheat lines expressing HMW-GS are not as stable as conventional breeding cultivars [42]. However, dough-mixing tolerance increases when *Glu-A1* is overexpressed [43]. In addition, dough quality improves when the expression level of the *Glu-D1* allele is maintained [43]. Previous studies have also demonstrated the importance of wheat HMW-GS, especially *Glu-A1* and *Glu D1*, in wheat quality breeding programs and bread-making quality.

In our peptide analysis, a total of 111 spots with differential expression under heat stress conditions were detected. These spots could be sorted into three groups based on their expression patterns (Figure S2). Glutenin spots showing decreased expression in group A (Figure S2) under heat stress were identified as Spot Nos. 5806, 6807, 7601, 7602, 8602, 8604 (Figure S2 and Figure 3, Table S1). The presence of glutenin subunits is known to be a genetically fixed qualitative trait. The presence or absence of certain subunits does not change, although the ratio of glutenin to gliadin decreases when the plant is exposed to high temperatures during grain filling [44]. This indicates that glutenin gene expression is more sensitive to heat stress than gliadin gene expression. Thus, we were interested in glutenin subunits which showed significant decreases in expression during heat stress. We selected six glutenin protein spots that showed significant decreases in expression in heat stressed plants. Protein sequence homology searches revealed four HMW-GS genes (*TraesCS1D02G317301*, *TraesCS1A02G317311*, *TraesCS1D02G317211*, and *Traes7B02G345400*) (Table 1). Of these, *TraesCS1D02G317301* was present in the highest frequency. The other genes (*TraesCS1A02G317311*, *TraesCS1D02G317211,* and *Traes7B02G345400*) showed low-level matches with putative proteins. *Traes7B02G345400* was found to be similar to an *Oryza sativa* storage protein. Therefore, the critically decreased spot identified from high-temperature-stressed plants was determined to be from the *TraesCS1D02G317301* (*TaHMW1D*) gene.

Several genes in wheat were revealed to deregulate through various transcript variants events. *TaHMW1D*, relative to HMW glutenin, exhibited transcript variants events during the grain filling stage under heat stress. We obtained full sequences of *TaHMW1D* and its four transcript variants (*TaHMW1D*-1, 2, 3, and 4) by performing PCRs using DAT10-C and DAT10-T cDNAs. *TaHMW1D* encoded a 69.68 kDa mature HMW glutenin, belonging to pronounced HMW-GSs with molecular masses ranging from 65 kDa to 90 kDa [14]. We also identified four *TaHMW1D* transcript variants of different sizes (*TaHMW1D*-1, 2, 3, and 4). Their frequencies increased during heat stress and in grains found at the edge of the spike (Figure 9). The expected protein sizes for transcript variants *TaHMW1D*-1, 2, 3, 4 were 38.38, 38.72, 36.54, and 32.32 kDa, respectively, all of which belong to the LMW-GS molecular mass range (~30–40 kDa). In other words, these findings show that *TaHMW1D* experiences deletion events under heat stress, generating several LMW-GS proteins that are similar in size (30–40 kDa). In a previous study, heat stress at the beginning of the grain filling period (16 days after anthesis) negatively affected HMW-GS and LMW-GS synthesis, resulting in a decreased HMW/LMW ratio [45]. One possible explanation for the decreased HMW/LMW ratio under heat stress is that the size of HMW glutenin decreases due to the increased production of the transcript variants. The ratio of HMW to LMW-GS could be a selection criterion leading to enhanced quality in wheat breeding programs. A number of *TaHMW1D* transcript variants might indicate another mechanism to regulate HMW-glutenin protein expression level under heat stress. However, the functional distinctions between these *TaHMW1D* transcript variants remain unclear.

When cDNA of the full CDS region was compared with gDNA, the *TaHMW1D* repetitive region was found to have only one exon (Figure 4 and Figure S3). All transcript variants revealed truncated proteins, maintaining HMW-GS function, by deletion a thet repetitive region within one exon. While the amino acid sequence of HMW-GS has a highly repetitive structure comprising different motifs, the HMW-GS gene, which is driven by a typical eukaryotic promoter, does not contain introns [46]. In studies on *Triticum aestivum*, analysis of the genomic regions and phylogenetic analysis of the α-gliadin gene have suggested the creation of diverse *A*, *B*, and *D* subgenomes through an evolutionary process [47]. Nascent allohexaploid wheat evolution with DNA deletions through hybridization among *Aegilops* and *Triticum* is known to contribute to structural and functional diversity by changing repetitive regions of the wheat genome [48]. The repetitive sequence of wheat has been recombined through duplication, insertion, inversion, and translocation in the process of evolution of several species, such that it now constitutes diverse repeated sequences. The repeated regions, which consisted of tandem repeats, could be useful functional regions which underwent rapid variation and diverse morphological plasticity in response to the environment [49]; although *TaHMW1D* has only one exon, repetitive sequences could easily be changed by evolutionary processes. These processes also frequently occur in response to stress.

*TaHMW1D* deletion events occurred only in repetitive sequences within the exon (Figure 8). Specific deletion sites and patterns in transcript variants were identified within an exon. Deletion events of pre-mRNA are known to occur only at specific deletion sites (5′ and 3′), branch-point sequences, and polypyrimidine tracts (which promote assembly of the spliceosome), all of which can serve as key signals in the repetitive region [50]. We found four transcript variants in the conserved trinucleotide sites GGG (glycine, G) or CAA (glutamine, Q) at the 5′ and 3′ ends of repetitive regions. The specific deletion sites and patterns identified in *TaHMW1D* transcript variants were novel in this paper. There were many 5′-CAAGG/TG-3′, 5′-GGGCAA-3′ sites in the repetitive regions (Table 2). The repetitive region located at the center of the full CDS sequence consisted of three types of sequence (tripeptides (GQQ), hexapeptides (PGQGQQ), and nonapeptides (GYYPTSLQQ)) in HMW-GS. The sequences differ between HMW-GS and LMW-GS [51]. Tripeptides and hexapeptides contain *TaHMW1D* specific deletion sites (GQ) (Table 2). We assume that many deletion events occur in the repetitive region. However, *TaHMW1D* also contains promoter and conserved regions (signal and N-/C-terminal peptides) in its CDS that do not exhibit sequence changes, suggesting that the spliceosome does not act on the conserved region, although specific deletion sites (GQ) are also present at the conserved sites (Figure 7 and Figure 8). We expect that the transcript variants of *TaHMW1D* may retain glutenin protein function, as they maintained conserved sites (signal and N-/C-terminal regions) and cysteine (five numbers in N terminal peptide and one number in C terminal peptide). Since all *TaHMW1D* transcript variants share the same HMW-GS repetitive region and conserved region, it is evident that the variants were derived from HMW-GS, not LMW-GS.

We found transcript variants that were generated by various sizes of expressions using different combinations the of repetitive regions within a *TaHMW1D* gene under heat stress during grain filling period. In a stressful environment, these events in repetitive regions can generate protein diversity to maintain homeostasis in the plant system. As one of the major high-molecular weight glutenin genes, *TaHMW1D* was found to undergo a different mode of transcription between the repetitive regions within the exon under high-temperature stress during an early stage of the grain filling. The plant chose to generate transcript variants that were smaller than full transcript of *TaHMW1D* but possess HMW glutenin properties as heat treatment progressed. Furthermore, the shorter protein products of the transcript variants retain the core functions of HMW-glutenin but sacrifice repetitive region(s) within the exon. Therefore, the transcript variants of *TaHMW1D* glutenin gene could serve as an active protective mechanism or a means of maintaining minimal gene function in a harsh environment. We expect that transcript variants events within the exon do not occur at random, but are instead associated with different gluten structures and heat-resistant properties in response to heat stress during the grain filling period. Although there are no known introns in glutenin genes, the removal of the repetitive regions under high-temperature stress during grain filling might act as a defense mechanism to minimize damage to glutenin production, as it allows the plants to keep producing shorter forms of glutenins while maintaining the characteristics of the HMW glutenins.

The transcript variants of *TaHMW1D* were especially regulated at mRNA level. The expression levels of the transcript variants were increased in distal parts of the spike after 10 days of heat treatment (DAT10-T#5). The grain yield and the weight per spike differ depending on spikelet position [9]. Although the grain size at the floret position in the spike is reduced by exposure to high temperatures and low light conditions, the grain size in the top spike is affected to a greater degree than that in lower positions under low irradiance. Abscisic acid (ABA), which regulated starch biosynthesis gene, was decreased under heat stress and changed relative to individual grains located at different spike positions in *Oryza sativa* [52]. The slower filling rates of the distal grains are known to be associated with abscisic acid, ethylene, and 1-aminocyclopropane-1-carboxylic acid levels [53]. In the same vein, the grain yield along the spike could depend on spikelet position. It is affected by stressful conditions. The cause of uneven grain development along the spike, especially under stressful conditions, has not yet been fully elucidated. Our results indicate that *TaHMW1D*, which encodes a seed storage protein, experiences various transcript variants events, especially in grains at distal parts of the spike, resulting in poor spikelet and grain development.

We found that the expression and translation of *TaHMW1D* was decreased by deletion events that occurred only in the repetitive regions at specific deletion sites. Gluten protein polymer structures are important factors in determining end-use quality. Under heat stress, plants shorten their grain-filling period and produce gluten polymers within a short period of time. Polymers are made up of several glutenins. Meanwhile, it is known that expression of the glutenin gene decreases, resulting in poor grain quality, under heat stress. However, this phenomenon might be expected to form grains by creating another short gluten polymer due to deletion events in the repetitive region. This novel finding could aid in addressing why the end-use quality of wheat grain declines under heat stress.

## 4. Methods

### 4.1. Plant Materials and Growth Conditions

The common wheat cultivar “*Keumgang*” (National Agrobiodiversity Centre, RDA, Korea; accession no. IT 213100) was used in this study. *Keumgang* seeds were vernalized at 4 °C for 5 weeks in the dark. After vernalization, each seedling was transferred to a pot (10 × 8 × 10 cm, top diameter x height x bottom diameter) filled with soil (Sunshine Mix #1, Sungro, USA). Plants were grown in phytotrons (Gaooze Control System, Suwon, Korea) set at 21 °C/16 °C for 16 h (600 µmol m^−2^ s^−1^)/8 h during the day/night. The anthesis time of the main stem in each plant was labeled. High-temperature stress (34 °C/31 °C, day/night) was applied to plants beginning 9 days after anthesis. Once the main spike of each plant reached a certain stage (9 days after anthesis), which was scored as starch development in watery kernel, the heat-treatment groups of plants were transferred to another phytotron with identical conditions except for temperature. Different groups of plants were subjected to two different heat stress periods, 6 and 10 days. The pots in the control and treatment phytotrons were randomly placed and rotated to avoid positional effects. After heat treatment, each grain from the base (#1) to the top (#5) of the spike (Figure 1A) was labeled as DAT6-C (6 days control treatment, Zadok scale 75), DAT6-T (6 days heat treatment, Zadok scale 75), DAT10-C (10 days control treatment, Zadok scale 77), and DAT10-T (10 days heat treatment, Zadok scale 77) for the transcriptional analysis (Figure 1B). For the proteomic analysis, heat-treated plants were moved to the control chamber and grown until harvest. After ripening, each spike was divided into five parts from the base. Grains were labeled Con (no heat treatment), DAT6-T (6 days heat treatment), and DAT10-T (10 days heat treatment) (Figure 1C). Three biological replicates were used.

### 4.2. Extraction and Fractionation of Gluten Protein

Total seed storage protein was extracted from seeds (Con, DAT6-T and DAF10-T, Figure 1C). Briefly, seeds were homogenized using a mortar and pestle. The resulting flour meal was suspended in a sample lysis solution (1 mL 70% ethanol) and incubated at 37 °C for 3 h with shaking (250 rpm). The mixture was centrifuged at 14,000 rpm at 4 °C. The supernatant was collected for gliadin. The precipitate was washed with washing buffer (50% (*v*/*v*) 1-propanal) and used for glutenin extraction. The remaining pellet was resuspended in 200 µL glutenin extraction buffer 1 (50% 1-propanol, 80 mM Tris-HCl (pH 8.0), 1% dithiothreitol (DTT)) and incubated at 65 °C for 30 min. After adding glutenin extraction buffer 2 (glutenin extraction buffer 1 with 1.4% 4-vinylpyridine), the mixture was incubated at 65 °C for 5 min. After centrifugation at 18,341× *g* for 10 min at 4 °C, the supernatant was collected for glutenin. The protein content was determined using the Bradford assay [54].

### 4.3. Sodium Dodecyl Sulfate-Polyacrylamide Gel Electrophoresis (SDS-PAGE)

SDS-PAGE analysis was performed to assess protein composition and intensity following a previously published protocol [55]. Total glutens were fractionated by SDS-PAGE for HMW-GS profiling. After the glutenin supernatant (1 mL) was removed by drying, 100 µL of 2X sample buffer [1.8% SDS, 12% glycerol, 54 mM Tris-HCl (pH 8.8), 1.8 mM EDTA (pH 8.0), and 0.006% bromophenol blue (BPB)] was added. After incubation at 65 °C for 15 min, 50 µL of sample was run on a 12% SDS-PAGE gel (30% acrylamide, 1.5 M Tris-HCl (pH 8.8), and 10% SDS) at 200 V for 18 h in a running buffer composed of 0.192 M glycine, 0.1% SDS, and 0.025 M Tris base. HMW-GSs were visualized on 12% SDS-PAGE gels (12% (*w*/*v*) acrylamide) and stained with staining solution (10% glacial acetic acid, 50% methanol, and 0.1% Coomassie Brilliant Blue R-250 (CBB)). The same solution without CBB was used to destain the gels.

### 4.4. Two-Dimensional Gel Electrophoresis (2DE)

2DE was performed for gluten separation by GENOMINE (Seoul, Korea). Briefly, IPG dry strips (4–10 NL IPG, 24 cm, Genomine, Korea) were equilibrated for 12–16 h with 7 M urea, 2 M thiourea containing 2% 3-[(3-cholamidopropy) dimethyammonio]-1-propanesulfonate (CHAPS), 1% dithiothreitol (DTT), and 1% Pharmalyte. IPG dry strips were loaded with 200 µg of gluten. Isoelectric focusing (IEF) was performed at 20 °C using a Multiphor II electrophoresis unit and an EPS 3500 XL power supply (Amersham Biosciences, Little Chalfont, UK) following the manufacturer’s instructions. For IEF, the voltage was increased linearly from 150 to 3500 V with focusing complete after 96 kVh. Before the second dimension, strips were incubated for 10 min in equilibration buffer (50 mM Tris-Cl, pH 6.8 containing 6 M urea, 2% SDS, and 30% glycerol) first with 1% DTT and then with 2.5% iodoacetamide. The equilibrated strips were then inserted onto SDS-PAGE gels (20 × 24 cm, 10–16%). SDS-PAGE was performed using a Hoefer DALT 2D system (Amersham Biosciences, Little Chalfont, UK). We ran the 2D gels at 20 °C and 1700 Vh. Separated protein fractions were silver-stained as described previously [56].

### 4.5. Image Analysis

Quantitative analysis of digitized images was done using PDQuest (version 7.0, BioRad, Hercules, CA, USA) following the manufacturer’s instructions. The quantity of each spot was normalized to total valid spot intensity. Protein spots with significant variations in expression were selected for further analysis.

### 4.6. Mass Spectrometry

MALDI-TOF was performed by GENOMINE (Korea). For protein identification by peptide mass fingerprinting, protein spots were excised, digested with trypsin (Promega, Madison, WI, USA), mixed with cyano-4-hydroxycinnamic acid in 50% acetonitrile with 0.1% trifluoroacetic acid, and subjected to matrix-assisted laser-desorption ionization time of flight (MALDI-TOF) analysis (Microflex LRF 20, Bruker Daltonics, Billerica, MA, USA). Spectra were collected from 300 shots per spectrum over a mass-to-charge (m/z) range of 600–3000. Two-point internal calibration was done using trypsin autodigestion peaks (m/z 842.5099, 2211.1046). A peak list was generated using Flex Analysis 3.0. Thresholds used to pick peaks were as follows: 500 for minimum resolution of monoisotopic mass and 5 for signal-to-noise (S/N). The search program MASCOT (version 2.1. Matrix Science, UK) was used for protein identification by peptide mass fingerprinting. The following parameters were used for the database search: trypsin as the cleaving enzyme, a maximum of one missed cleavage, iodoacetamide as a complete modification, oxidation as a partial modification, monoisotopic masses, and a mass tolerance of ± 0.1 Da. The peptide mass fingerprinting (PMF) acceptance criterion was based on probability scoring. Protein identification was carried out using MASCOT. Searches were performed against NCBI’s nonredundant protein database and calculated using MASCOT. A minimum sequence coverage of 25% was used.

### 4.7. Genomic DNA Extraction

Total genomic DNA was extracted from control seeds (Con) using cetyltrimethylammonium bromide (CTAB) [57]. The quality and concentration of DNA were estimated using a Nanodrop-2000 spectrophotometer.

### 4.8. RNA Extraction and cDNA Synthesis

Dehulled spikelets (DAT6-C, DAT6-T, DAT10-C, and DAT10-T, Figure 1B) from three replicate plant samples were used for transcriptional analysis. “DNA-free seed RNA extraction” [58] was used to extract total RNA. RNA concentration was measured using a Nanodrop-2000 spectrophotometer. RNA integrity was estimated using 1.0% agarose gel electrophoresis. All RNA samples were adjusted to the same concentration to standardize the RNA input for subsequent reverse transcription reactions. First-strand cDNAs were synthesized from 1 µg of total RNAs in a volume of 20 µL per reaction using a PrimeScript 1st strand cDNA Synthesis Kit (Takara, Seoul, Korea) following the manufacturer’s protocol. cDNAs were diluted to a final volume of 100 µL for PCR.

### 4.9. Isolation and Sequencing of a Wheat HMW-GS Genes

To identify putative genes, the sequences of HMW glutenin genes were amplified using primers (forward: 5′-CAACCAATCTCCACAATTTCA-3′ and reverse: 5′-GTGGGTCATCAATATGCATCAACA-3′) designed based on a wheat data Blast search (http://plants.ensemble.org/Triticum_aestivum/Info/Index (accessed on 20 November 2017)). PCR amplification was performed with a high-fidelity i-pfu DNA polymerase (IntRON, Seongnam, Korea). The PCR program consisted of a denaturation step at 95 °C for 3 min, 38 cycles of 94 °C for 30 s, 58 °C for 10 s, and 72 °C for 2 min, with a final extension step at 72 °C for 3 min. After purification using a FavorPrep GEL/PCR purification mini KIT (Favorgen, Seoul, Korea), the PCR products were cloned into a PLUG-Prime TA-Cloning vector KIT II (IntRON, Seongnam, Korea) and introduced into ECOS 101 Competent Cells (DH5-α, Biotech, Seongnam, Korea) as described previously (Sambrook and Fritsch 1989). The resulting sequences were analyzed using NCBI blast (https://blast.ncbi.nlm.nih.gov/ (accessed on 22 January 2019)), the Ensembl plant database (https://plants.ensembl.org/Multi/Tools/Blast (accessed on 15 November 2017)), and URGI (https://urgi.versailles.inra.fr/blast/ (accessed on 17 August 2018)). Repeated sequences were found using the Repeats Finder for DNA/Protein Sequences (https://www.novoprolabs.com/tools/repeats-sequences-finder (accessed on 1 January 2013)).

### 4.10. Quantitative Reverse Transcription-Polymerase Chain Reaction (qRT-PCR) Analysis

Primers amplifying conserved regions (CONS-F (5′- TCCTCTTTGCGGCAGTAGTC -3′), CONS-R (5′-CTTGGCGCTAACATCTCGGA-3′), located in signal and N-terminal peptide, present in complete *TaHMW1D* and its transcript variants) and deleted regions (REP1-F (5′-GGGCAAATCCCAGCTTCT-3′), REP1-R (5′-CCTTTGTCCTGGCTGTCCTT-3′), located at repetitive region 4) were used (Figure 8). PCR primers were designed using NCBI primer-BLAST (https://www.ncbi.nlm.nih.gov/tools/primer-blast/ (accessed on 19 June 2018)). qRT-PCR was conducted using cDNA in a 10 µL mixture containing BrightGreen 2X qPCR MasterMix (abm, Canada). Initial denaturation was done at 95 °C for 10 min, which was followed by 40 cycles of 95 °C for 15 s, 57 °C for 1 min, 72 °C for 10 s, and a final extension at 72 °C for 5 min. Three replicates (biological and technical) were run for each stage.

### 4.11. Statistical Analyses

The qRT-PCR experiments were repeated three times under the same conditions. Analysis of variance (ANOVA) was performed using SPSS11.0 (SAS Institute Inc., Cary, NC, USA) followed by Duncan’s multiple-range test, where *p* < 0.05 (*) or *p* < 0.001 (***) was considered significant.

## 5. Conclusions

Wheat produce *TaHMW1D* transcript variants within a short period of time for seed development, for example, as a way of avoiding disturbance to grain constituents under heat stress as well as in the distal position of the spike. This transcript variants event always occurred between specific sequence sites at repetitive regions in an exonic region. Although the functional distinctions between these *TaHMW1D* transcript variants remain unclear, considering the importance of HMW-GS as grain storage proteins, stressed plants may preferentially make short polypeptides while retaining glutenin functionality, motifs as well as conserved sites.

## Figures and Tables

**Table 1:** Protein identification by MALDI-TOF mass spectrometry. Protein name, identification number (NCBI data); protein mass, the expected mass of a protein with a high-confidence identity match; total matching score, combined score of all observed mass spectra that can be matched to amino acid sequences; matches number, the number of MS/MS spectra matched to the protein; protein information, name of genes with significant alignment; highest identity, the highest percent identity between a set of aligned segments and the same subject sequence; identity (%), the percent of query length that is included in the aligned segments.

Protein Name	Protein Mass (Da)	Total Matching Score	Matches Number	Protein Information (NCBI Data)	Highest Identity (Plants Ensemble)	Identity (%)
AAF37838.1	19,968	120	11	y-type high molecular weight glutenin subunit, partial [Aegilops ventricosa]	TraesCS1D02G317301	100
AAP73788.1	37,935	64	8	y-type HMW glutenin subunit, partial [Triticum spelta]	TraesCS1D02G317301	100
AAP73790.1	37,940	64	8	y-type HMW glutenin subunit, partial [Triticum compactum]	TraesCS1D02G317301	100
AAR04373.1	47,810	57	8	high molecular weight glutenin subunit type y [Aegilops tauschii]	TraesCS1D02G317301	99.30
AAT06762.1	27,382	124	12	HMW glutenin subunit Dy10 [Aegilops tauschii]	TraesCS1D02G317301	100
ABF82252.1	89,645	223	25	high molecular weight glutenin subunit [Triticum aestivum]	TraesCS1D02G317301	93
ABK54365.1	88,693	82	9	high molecular weight glutenin subunit [Triticum aestivum]	TraesCS1D02G317301	100
ABN71647.1	32,308	68	8	truncated high molecular weight glutenin subunit 1By9 [Triticum aestivum subsp. tibeticum]	TraesCS1D02G317301	100
ACL82342.1	17,566	125	11	high molecular weight glutenin y-type, partial [Triticum aestivum]	TraesCS1D02G317301	99.30
ACZ49742.1	91,648	328	31	high molecular weight glutenin subunit Ax-dp, partial [Triticum polonicum]	TraesCS1A02G317311	99.10
ADY38692.1	90,025	258	27	high-molecular-weight glutenin subunit [Secale cereale x Triticum aestivum]	TraesCS1D02G317301	92.3
ADY38693.1	66,498	121	14	high-molecular-weight glutenin subunit [Secale cereale x Triticum aestivum]	TraesCS1D02G317301	92.3
ADY38695.1	65,788	122	14	high-molecular-weight glutenin subunit [Secale cereale x Triticum aestivum]	TraesCS1D02G317301	92.3
ADY38698.1	64,245	123	14	high-molecular-weight glutenin subunit [Secale cereale x Triticum aestivum]	TraesCS1D02G317301	99.3
ADY38699.1	66,442	122	14	high-molecular-weight glutenin subunit [Secale cereale x Triticum aestivum]	TraesCS1D02G317301	92.3
ADY38701.1	63,448	124	14	high-molecular-weight glutenin subunit [Secale cereale x Triticum aestivum]	TraesCS1D02G317301	92.3
ADY38705.1	64,840	296	27	high-molecular-weight glutenin subunit [Secale cereale x Triticum aestivum]	TraesCS1D02G317301	90.9
ADY38706.1	66,308	293	27	high-molecular-weight glutenin subunit [Secale cereale x Triticum aestivum]	TraesCS1D02G317301	92.3
ADY38717.1	63,738	124	14	high-molecular-weight glutenin subunit [Secale cereale x Triticum aestivum]	TraesCS1D02G317301	92.3
ADY38718.1	64,758	209	22	high-molecular-weight glutenin subunit [Secale cereale x Triticum aestivum]	TraesCS1D02G317301	92.3
AEO19857.1	93,820	254	27	high molecular weight glutenin subunit [Triticum aestivum]	TraesCS1D02G317301	99.3
AHZ62762.1	90,009	258	27	high molecular weight glutenin subunit 1Ax1 [Triticum aestivum]	TraesCS1A02G317311	100
AKW50839.1	90,741	300	29	high molecular weight glutenin subunit [Triticum aestivum]	TraesCS1D02G317301	98
AKW50840.1	90,254	126	16	high molecular weight glutenin subunit [Triticum dicoccoides]	TraesCS1D02G317301	100
AKW50841.1	90,291	126	16	high molecular weight glutenin subunit [Triticum aestivum]	TraesCS1D02G317301	100
AKW50842.1	90,042	209	24	high molecular weight glutenin subunit [Triticum aestivum]	TraesCS1D02G317301	100
ANJ03342.1	90,244	136	16	HMW-GS protein [Triticum dicoccoides]	TraesCS1D02G317301	100
BAJ85955.1	13,150	52	4	predicted protein [Hordeum vulgare subsp. vulgare]	No match	
BAN29068.1	86,558	335	31	high molecular weight glutenin subunit, partial [Triticum aestivum]	TraesCS1D02G317211	100
BAN82580.1	89,546	223	25	high-molecular-weight glutenin subunit [Triticum aestivum]	TraesCS1D02G317301	100
BAS99197.1	8,082	52	3	Os06g0686900 [Oryza sativa Japonica Group]	TraesCS7B02G345400	86.40
CAA43331.1	89,993	258	27	high molecular weight glutenin subunit 1Ax1 [Triticum aestivum]	TraesCS1A02G317311	99
CAC84118.1	21,859	198	16	glutenin high molecular weight subunit, partial [Triticum aestivum]	TraesCS1D02G317301	100
CAC84120.1	20,193	56	4	glutenin high molecular weight subunit, partial [Triticum aestivum]	TraesCS1D02G317301	98.60
EAZ38071.1	7,841	53	3	hypothetical protein OsJ_22417 [Oryza sativa Japonica Group]	TraesCS7B02G345400	86.40
EMS67071.1	90,299	108	13	Glutenin, high molecular weight subunit DX5 [Triticum urartu]	TraesCS1D02G317301	100
POF00734.1	14,483	54	4		No match	
XP_002879350.1	16,644	52	4	uncharacterized protein LOC9317273 [Arabidopsis lyrata subsp. lyrata]	No match	
XP_007513787.1	26,443	53	4	hypothetical protein Bathy04g00280 [Bathycoccus prasinos]	No match	
XP_020873876.1	16,658	52	4	uncharacterized protein LOC9299019 [Arabidopsis lyrata subsp. lyrata]	No match	

**Table 2:** The most abundant repeat sequences in *TaHMW1D* (sequences that experience specific deletion sites after high-temperature stress are underlined).

Repeated Sequence	Position	Length
AGCTTCTCAGCAGCAGCCAGGACAAGGGCAACAAGGGCACTACCCAGCTTCTCAGCAGCAGCCAGGACAAGGGCAACAAGGGCACTACCCAGCTTCTCAGCA	844	102
CAGGACAAAGGCAACAACCAGGACAAGGGCAACATCCAGAACAAGGG	320, 1082	47
AAGGGCAACAAGGGTACTACCCAACTTCTCTGCAGCA	236, 281	37
AGGACAAGGGCAACAAGGGTACTACCCAACTTCTCT	276, 978	36

## Data Availability

This research did not report any data.

## Supplementary Materials

The following are available online at https://www.mdpi.com/article/10.3390/plants10040687/s1, Figure S1: 2DE profiles of total seed storage proteins. Figure S2: 2DE expression patterns. Figure S3: Identification of *TaHMW1D* using genomic DNA. Figure S4: DNA sequence alignment of *TaHMW1D* in *Keumgang.* Table S1: Spots that showed decreased protein levels after high-temperature stress during grain filling.
