# Supplementary material for: Wheat (Triticum aestivum L.) TaHMW1D Transcript Variants Are Highly Expressed in Response to Heat Stress and in Grains Located in Distal Part of the Spike
Source: Plants (Basel). 2021 Apr 2;10(4):687. doi: 10.3390/plants10040687 (PMC8065890; doi:10.3390/plants10040687)
Supplement: Supplementary file 1 [file plants-10-00687-s001.zip › SUPPLEMETARY RESUBMITTED/Supplementary Figure 1.pdf]

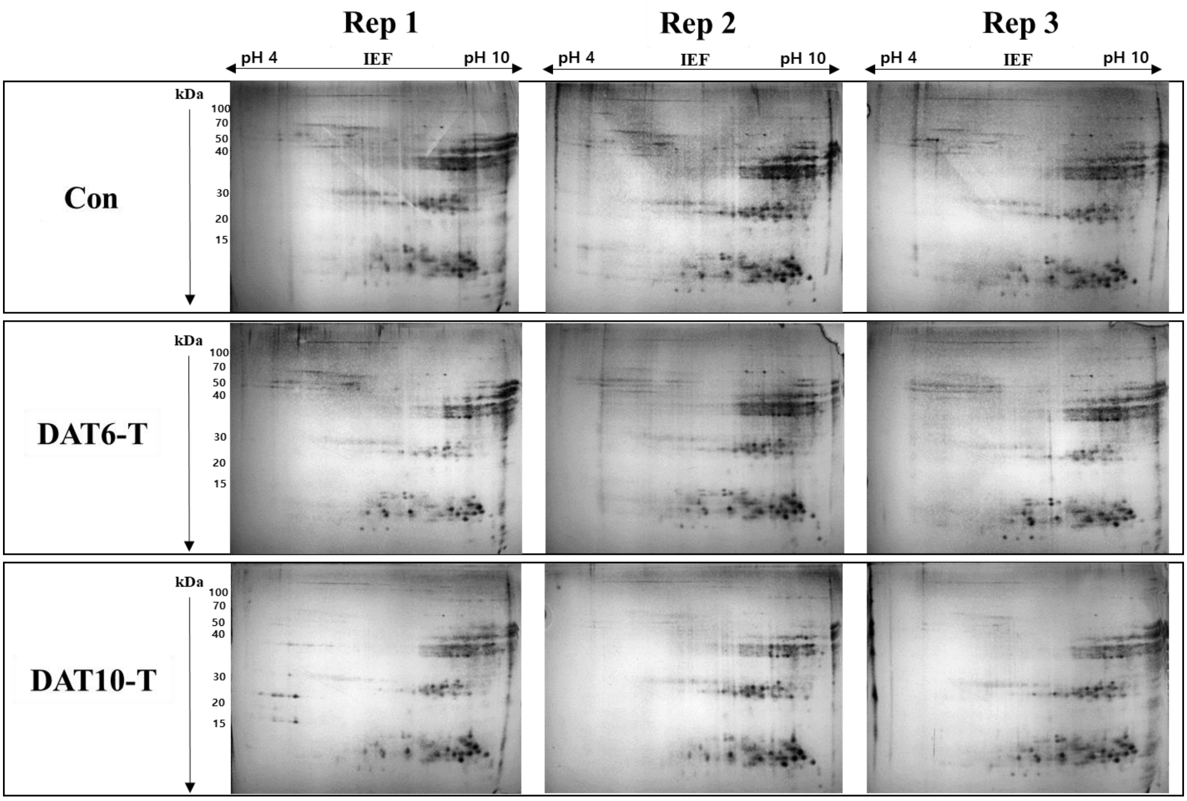

Figure S1. 2DE profiles of total seed storage proteins. PI ranges are displayed at the top. Protein sizes are displayed to the left. Con, non-treated control; DAT6-T, 6 days of treatment; DAT10-T, 10 days of treatment. Three biological replicates were used (Rep. 1 – 3).
