# Supplementary material for: Wheat (Triticum aestivum L.) TaHMW1D Transcript Variants Are Highly Expressed in Response to Heat Stress and in Grains Located in Distal Part of the Spike
Source: Plants (Basel). 2021 Apr 2;10(4):687. doi: 10.3390/plants10040687 (PMC8065890; doi:10.3390/plants10040687)
Supplement: Supplementary file 1 [file plants-10-00687-s001.zip › SUPPLEMETARY RESUBMITTED/Supplementary Figure 3.pdf]

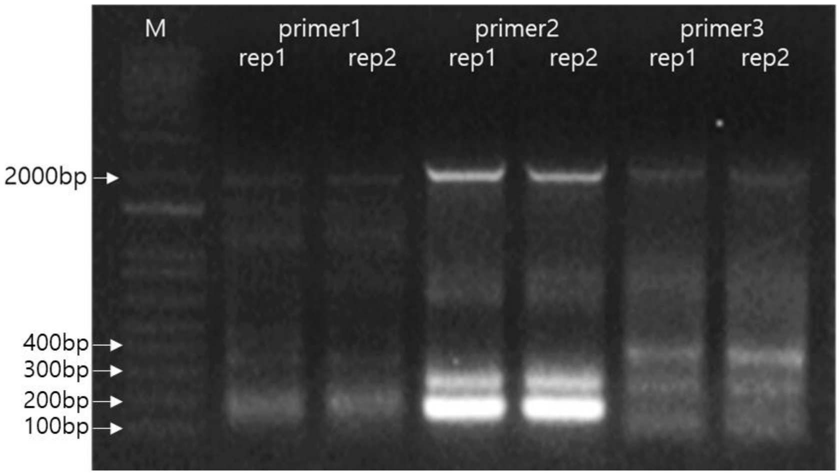

Figure S3. Identification of *TaHMW1D* full length using genomic DNA. Agarose gel (1%) showing the 2242 bp *TaHMW1D* gene product.
