# Supplementary material for: Wheat (Triticum aestivum L.) TaHMW1D Transcript Variants Are Highly Expressed in Response to Heat Stress and in Grains Located in Distal Part of the Spike
Source: Plants (Basel). 2021 Apr 2;10(4):687. doi: 10.3390/plants10040687 (PMC8065890; doi:10.3390/plants10040687)
Supplement: Supplementary file 1 [file plants-10-00687-s001.zip › SUPPLEMETARY RESUBMITTED/Supplementary Table 1.pdf]

Table S1. Spots that showed decreased protein levels after high-temperature stress during grain filling

SSP No.	Control	DAT6-T	DAT10-T	Protein
	Intensity (log2)			
7601	9.774687	9.676116	9.656116	
7602	11.89874	10.75452	10.20216	
8602	13.16445	12.96445	12.43546	glutenin
8604	11.85698	10.98567	10.58236	
5806	10.00486	9.97303	9.954172	
6807	10.438306	10.119806	9.747378	
3402	11.97937	11.97937	11.94354	
2403	11.751	11.65999	11.58892	
2402	11.14674	10.67069	10.28957	
6302	11.403	11.35457	10.58631	
5402	11.31695	11.26874	10.94575	
5301	11.46622	11.32659	11.09173	Alpha-,Beta-gliadin
5202	12.16641	12.08119	11.63084	
3301	11.24385	11.16644	10.88788	
2302	11.77691	11.56813	11.12797	
1301	10.16227	10.14391	9.425538	
2402	11.14674	10.67069	10.28957	
5002	13.12194	13.11979	13.06455	
4202	11.64567	11.64567	11.59977	
3101	11.81317	11.70669	11.70127	
2203	12.76997	12.66929	12.64088	
1101	11.32943	11.17591	11.15075	
1103	11.7182	11.66809	11.56589	
4203	9.444297	9.444297	9.36724	
1002	9.057541	9.00393	8.94825	
6203	11.20835	11.16966	11.06731	non-gluten proteins
7101	12.52412	12.42856	12.1375	
1	8.74461	8.58297	8.566485	
7201	12.73685	12.72269	12.50416	
7102	12.35124	12.22713	11.43226	
6202	11.57788	11.54463	11.27134	
3202	10.19053	10.19053	9.811016	
2001	10.65998	10.65998	10.50314	
7103	8.647746	8.597084	8.102903	
7101	12.52412	12.42856	12.1375	
