# Supplementary figures and images for: Wheat (Triticum aestivum L.) TaHMW1D Transcript Variants Are Highly Expressed in Response to Heat Stress and in Grains Located in Distal Part of the Spike
Source: Plants (Basel). 2021 Apr 2;10(4):687. doi: 10.3390/plants10040687 (PMC8065890; doi:10.3390/plants10040687)

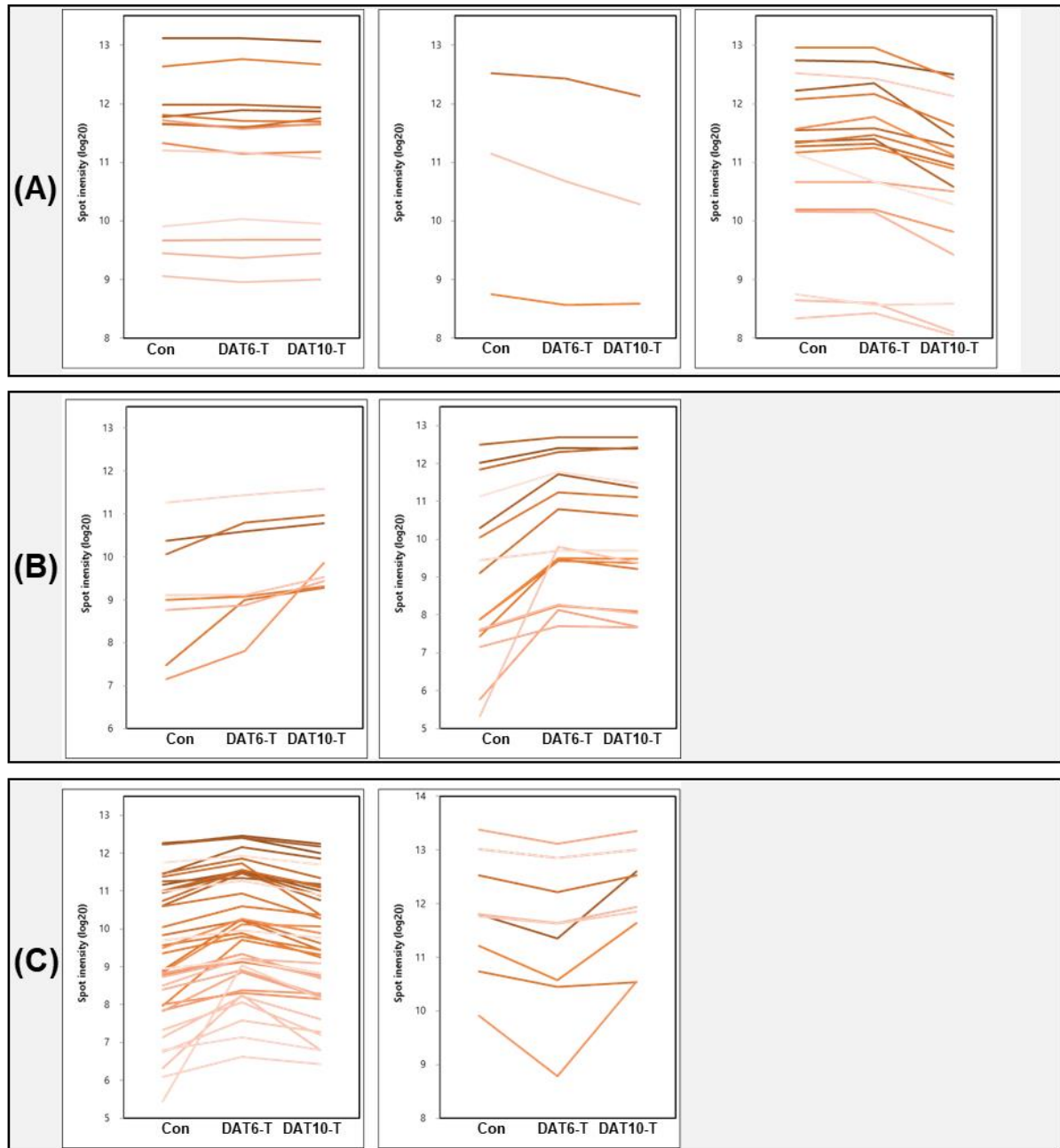

**Figure S2.** 2DE expression patterns

Supplement: Supplementary file 1 [file plants-10-00687-s001.zip › SUPPLEMETARY RESUBMITTED/Supplementary Figure 2.pdf]
