# Supplementary material for: Wheat (Triticum aestivum L.) TaHMW1D Transcript Variants Are Highly Expressed in Response to Heat Stress and in Grains Located in Distal Part of the Spike
Source: Plants (Basel). 2021 Apr 2;10(4):687. doi: 10.3390/plants10040687 (PMC8065890; doi:10.3390/plants10040687)
Supplement: Supplementary file 1 [file Supplementary Figure 4.pdf › SUPPLEMETARY RESUBMITTED/Supplementary Figure 4.pdf]

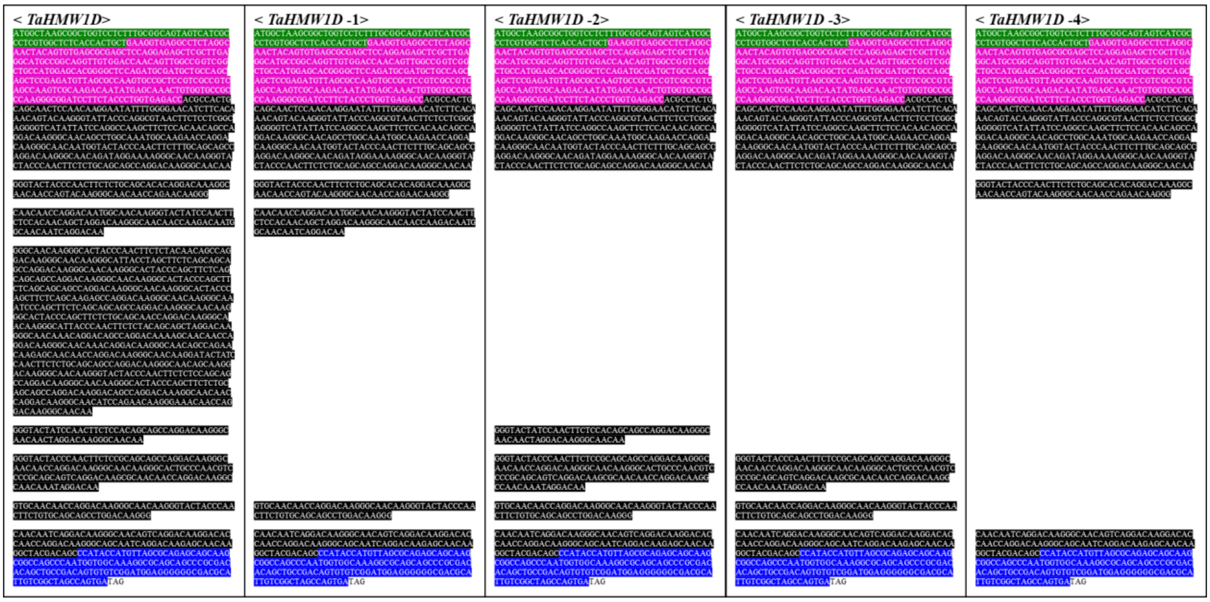

Figure S4. DNA sequence alignment of *TaHMW1D* in *Keumgang*.
